# Favorable aortic remodeling following type B aortic dissection in a patient with osteogenesis imperfecta

**DOI:** 10.1016/j.jvscit.2026.102400

**Published:** 2026-07-03

**Authors:** Charles Davis, Kameel Khabaz, Matheus Martino-Wojciechowski, Luka Pocivavsek

**Affiliations:** a Section of Vascular Surgery, Department of Surgery, University of Chicago, Chicago, IL; b David Geffen School of Medicine at UCLA, Los Angeles, CA

**Keywords:** Osteogenesis imperfecta, Aortic dissection, Thoracic aorta, Aortic remodeling, Connective tissue disorder

## Abstract

Osteogenesis imperfecta is a heritable type I collagen disorder, with aortic dissection a recognized complication. A 69-year-old man with osteogenesis imperfecta presented with acute uncomplicated type B aortic dissection with a thrombosed false lumen, managed with anti-impulse therapy targeting systolic blood pressure < 120 mm Hg and heart rate < 70 beats/min. Despite an ulcer-like projection and 4.2 cm proximal descending aortic diameter, serial imaging demonstrated complete resolution with optimal medical therapy by 6 months and reduction to 3.2 cm at 12 months. This favorable remodeling is notable given the underlying collagen disorder, where tissue fragility raises concerns about impaired healing and aortic integrity.

Osteogenesis imperfecta (OI) is a heritable connective tissue disorder predominantly caused by autosomal dominant mutations in collagen type I alpha 1 (COL1A1) and collagen type I alpha 2, which encode type I collagen.^1^^,^^2^ Although OI is classically recognized for skeletal manifestations including recurrent fractures and bone deformities, cardiovascular complications represent an increasingly important and potentially life-threatening aspect of the disease.^2^, ^3^, ^4^ Type I collagen provides essential tensile strength to the aortic wall, with mutations compromising extracellular matrix integrity.^5^^,^^6^ Aortic aneurysms and dissections have been reported in 10% to 30% of patients with OI.^5^ Despite growing recognition of aortic root dilatation and valvular dysfunction, aortic dissection remains a rare but underappreciated complication.^7^^,^^8^

Type B aortic dissection (TBAD) carries a guarded long-term prognosis, with aneurysmal degeneration in 60% to 70% of medically managed patients and late mortality of 25% to 30% at 3 to 5 years.^9^, ^10^, ^11^ Guidelines recommend hemodynamic control as first-line therapy, with thoracic endovascular aortic repair reserved for high-risk or complicated cases.^12^ Medical management alone rarely achieves favorable aortic remodeling (19.4% in the Investigation of Stent Grafts in Aortic Dissection [INSTEAD] trial).^9^^,^^10^

The authors present a 69-year-old man with OI developing an acute uncomplicated TBAD. Despite his collagen disorder, the patient demonstrated complete resolution of the dissection on serial imaging over a 12-month period. This case highlights the potential for favorable remodeling in select patients managed with optimal medical therapy alone. The patient provided written consent for the publication of their case details and images.

## Case presentation

A 69-year-old man with OI presented with acute onset of a popping sensation in his chest and abdomen radiating to the right arm, with no associated neurological deficits or signs of malperfusion. Computed tomography angiography (CTA) revealed an acute uncomplicated TBAD with a thrombosed false lumen radiographically identified as a crescentic intramural hematoma (IMH). Abdominal CTA also showed beaded bilateral external iliac arteries with a focal left external iliac artery dissection. Additional medical history was notable for genetically confirmed type I OI, hypertension, obstructive sleep apnea with continuous positive airway pressure noncompliance, and a history of 33 previous fractures. Family history was significant for a father with aortic aneurysm and a son with intracranial hemorrhage. Laboratory evaluation was notable for a creatinine of 1.42 mg/dL (estimated glomerular filtration rate 53.5 mL/min/1.73 m^2^), C-reactive protein of 0.32 mg/dL, and erythrocyte sedimentation rate of 1.0 mm/h. The patient was started on an intravenous esmolol drip targeting systolic blood pressure below 120 mm Hg and heart rate below 70 beats/min and admitted to the intensive care unit. No indications for acute surgical or endovascular intervention were identified. After stabilization, the patient was transitioned to oral valsartan and metoprolol succinate, with follow-up blood pressures consistently below 130/75 mm Hg and heart rates below 85 beats/min.

One month after presentation, symptoms and blood pressure were well-controlled. CTA demonstrated crescentic hypoattenuating thickening extending from the mid-aortic arch just beyond the left common carotid artery origin to the abdominal aorta, consistent with the largely thrombosed false lumen. Multiple foci of contrast pooling were identified within the crescentic thickening, with the most proximal focus demonstrating a wide-mouth communication with the true lumen, consistent with an ulcer-like projection measuring 7.9 × 12.1 mm with a depth of 3.5 mm. The proximal descending aorta measured up to 4.2 cm, whereas the mid ascending aorta measured 3.8 cm.

**Fig 1:**
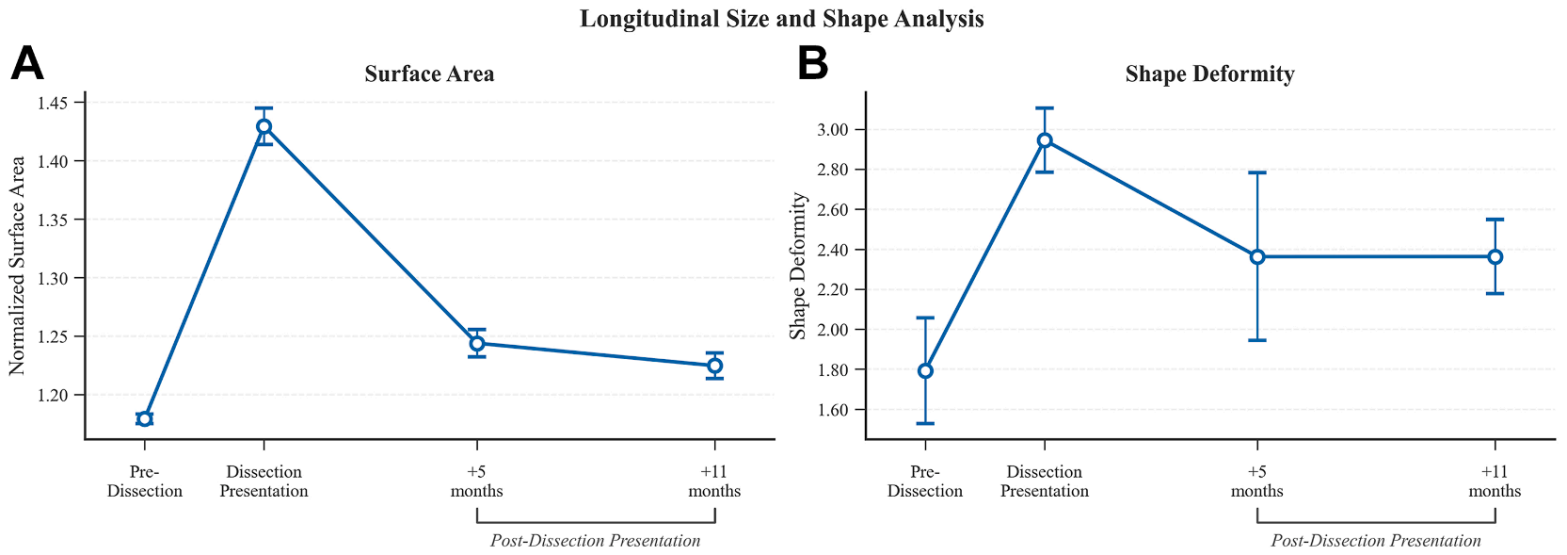
Geometric analysis of patient thoracic aorta at clinically relevant time points. Medical image segmentations of the patient's thoracic aorta were generated. Segmentations were then used to generate a surface mesh representation of the thoracic aorta. Thoracic aorta surface area and shape deformation was then calculated for four separate clinically relevant time points and plotted. **A,** Thoracic aorta surface area over time. **B,** Thoracic aorta shape deformation over time. *Error bars* indicate variance associated with different mesh resolution and mesh partitioning parameters.

**Fig 2:**
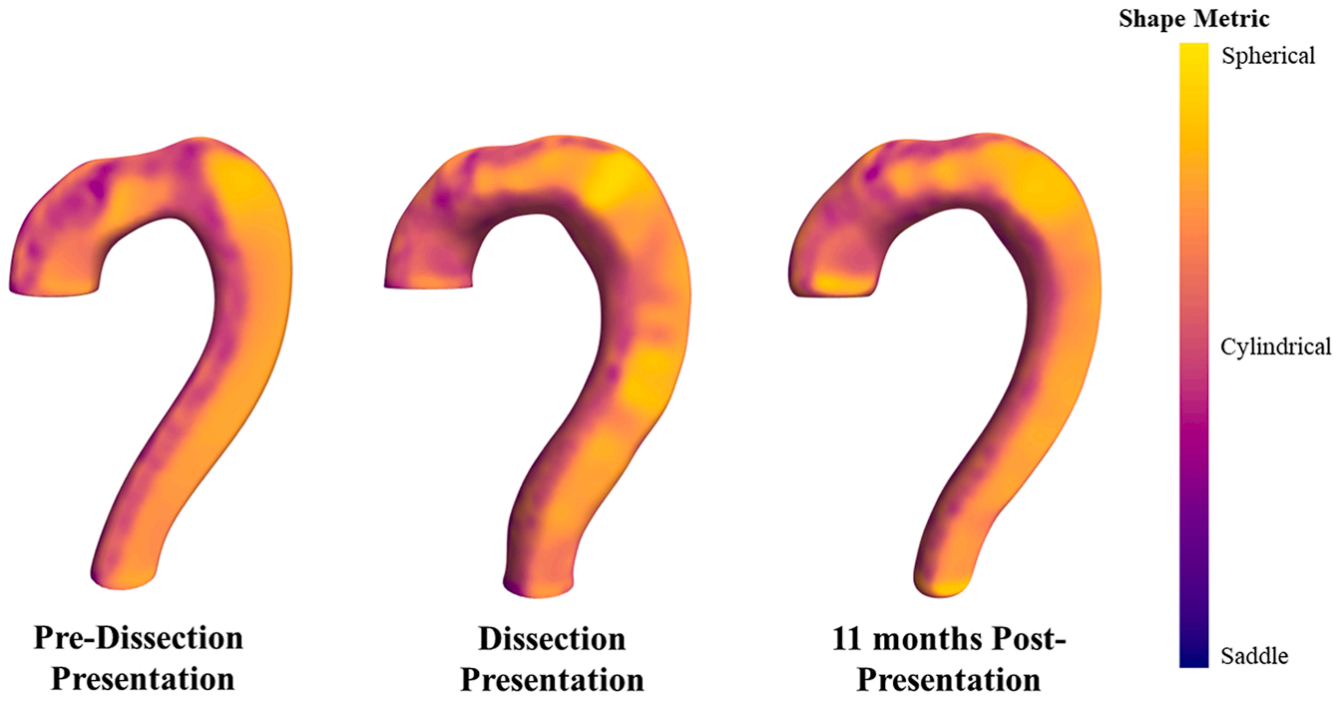
Shape metric visualized on patient aortic geometry. For three clinically relevant timepoints, shape metric (Koenderink Shape Index) is plotted as a color axis on top of a segmentation of the patient's thoracic aorta. *Darker, more purple colors* indicate more saddle-like shape regions, whereas *lighter, yellow colors* indicate sphere-like shape regions. The Koenderink Shape Index is a dimensionless, scale-independent measure of local surface curvature ranging from −1 (concave) to +1 (convex), with intermediate values representing saddle-like geometries. In the context of aortic morphology, a normal aorta exhibits a narrow distribution of shape index values consistent with a smooth cylindrical surface, whereas dissection introduces increased heterogeneity in shape index across the aortic wall.

**Fig 3:**
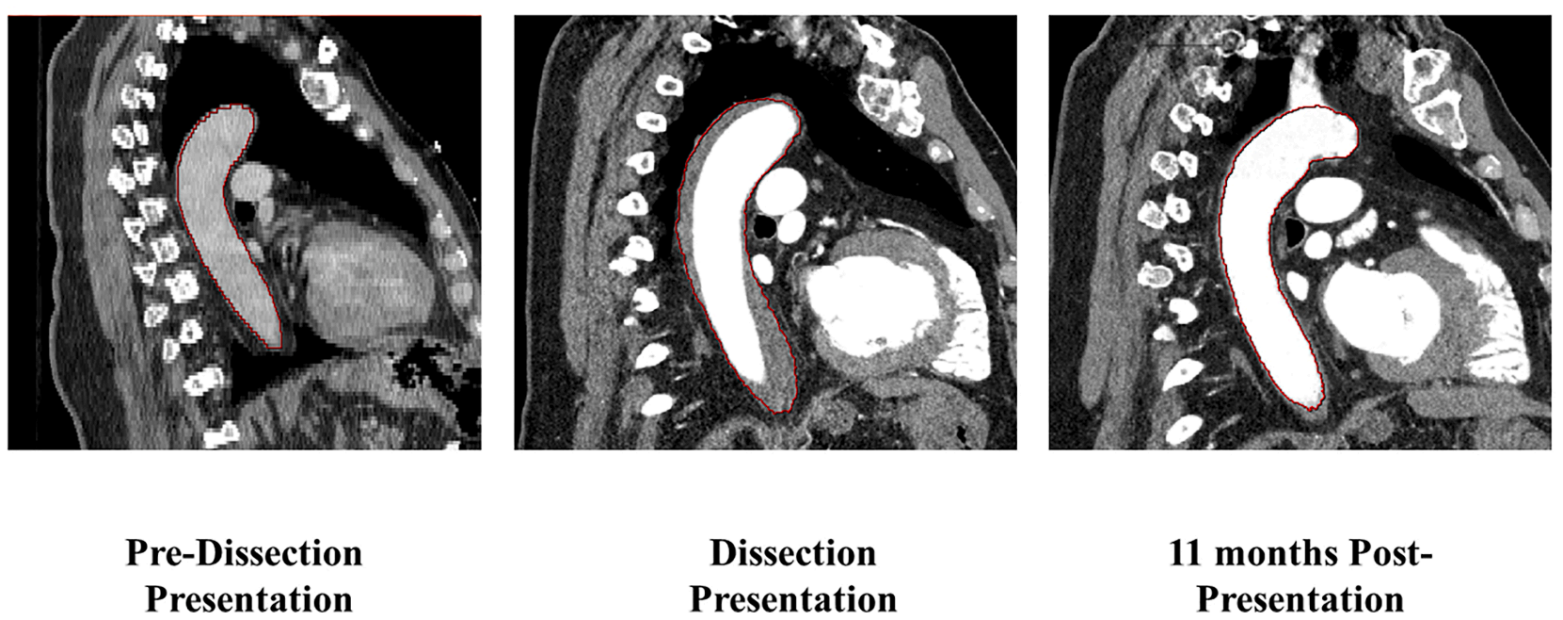
Computed tomography (CT) image data evolution. Sagittal view of patient CT image data for three clinically relevant timepoints. *Red* outline indicates boundaries of thoracic aorta segmentation.

**Fig 4:**
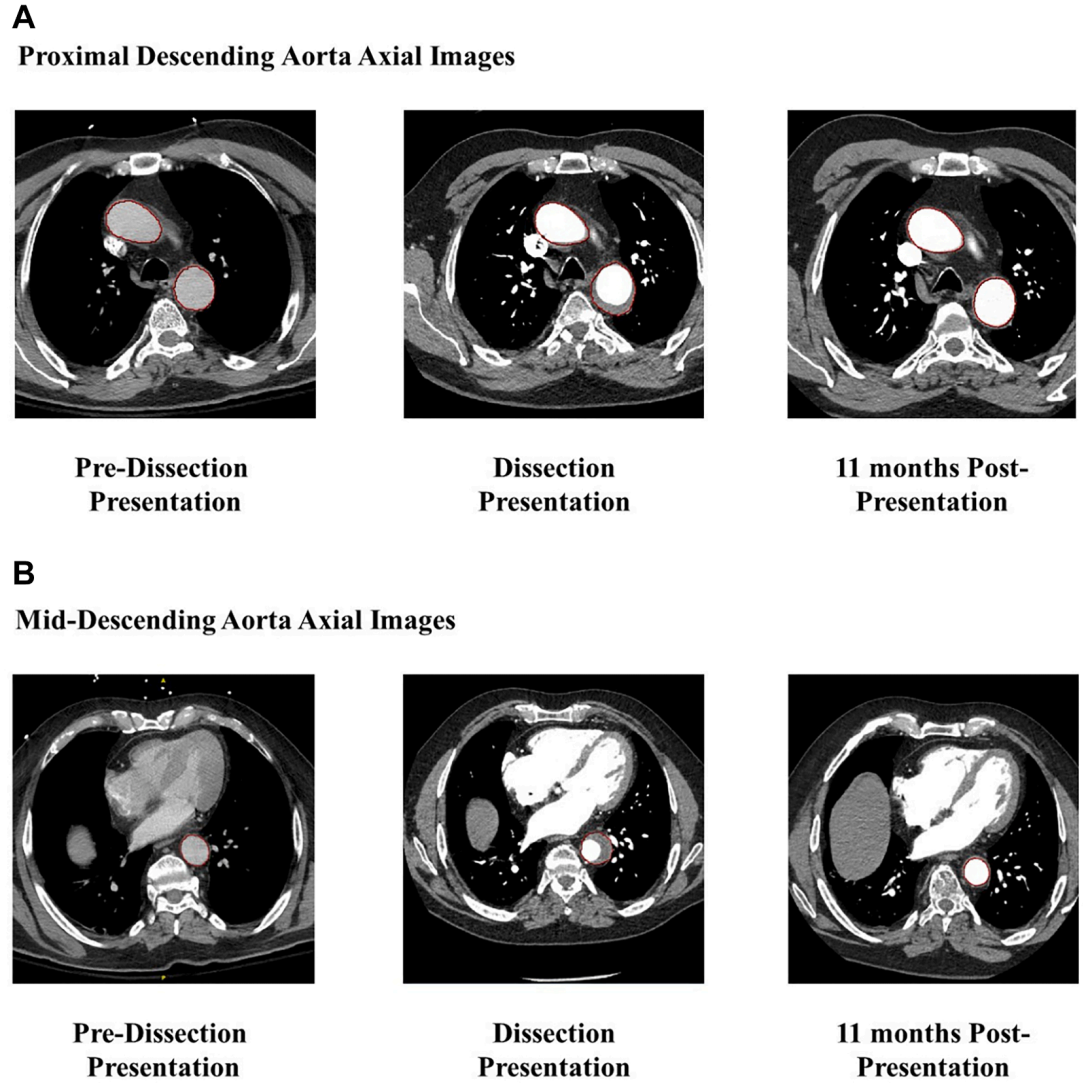
Axial computed tomography angiography (CTA) images of the aorta at two anatomic levels across three time points. **A,** Proximal descending aorta at predissection, dissection presentation, and 11 months postpresentation. **B,** Mid descending aorta at the same time points. Progressive resolution of the thrombosed false lumen and restoration of aortic wall morphology is demonstrated.

Approximately 6 months postpresentation, the patient remained well-controlled, and CTA demonstrated interval resolution of the hypoattenuating crescentic thickening and all previous foci of contrast pooling. The proximal descending aorta had decreased in size from 4.2 to 3.6 cm. The abdominal IMH had resolved, and the patent abdominal aorta showed no evidence of aneurysm, dissection, or residual IMH. At 1 year, CTA showed further resolution, with proximal descending aortic diameter decreasing to 3.2 cm. Serial geometric analysis demonstrated progressive improvement in aortic surface area and shape deformity following dissection presentation, with both measures declining substantially, though neither returned fully to predissection baseline despite favorable diameter reduction (Fig 1). Local surface curvature analysis demonstrated disruption of aortic geometry at dissection presentation with partial recovery by 11 months (Fig 2). Sagittal CTA confirmed complete resolution of the thrombosed false lumen with marked improvement in aortic wall morphology (Fig 3). Representative axial CTA images at the proximal and mid descending aorta demonstrate the initial pathology and subsequent resolution (Fig 4).

## Discussion

This case illustrates complete resolution of an acute uncomplicated TBAD with a thrombosed false lumen in a patient with OI. OI is increasingly recognized as a multisystem disorder with cardiovascular implications, most commonly valvulopathies and aortic root dilatation.^8^ The incidence of aortic dissection in OI remains poorly characterized, with a recent review reporting only six cases.^7^ Among these, previous reported cases involved surgical intervention, postoperative mortality, or both, and the present case adds a distinct outcome of favorable aortic remodeling with medical therapy alone.^7^ Case series and cohort data have documented life-threatening vascular complications, including dissections, in a substantial proportion of patients with COL1-related disorders.^5^^,^^6^ Against this backdrop, complete resolution of this dissection, despite conventional risk factors including hypertension and a family history of vascular disease, with optimal medical therapy alone is not well-characterized in the literature. Quantitative geometric analysis provided objective corroboration, demonstrating progressive improvement in aortic surface area and shape deformity (Fig 1; sensitivity to mesh resolution and partitioning parameters is reflected in error bars). These geometric metrics, previously validated for quantitative aortic morphologic analysis,^13^ complement standard diameter-based surveillance by capturing three-dimensional morphologic changes not reflected by diameter alone.

Medically managed uncomplicated TBAD carries a guarded prognosis, with aneurysmal degeneration exceeding 60% and survival below 50% in some series.^9^^,^^10^ False lumen thrombosis, a key marker of favorable aortic remodeling, is achieved in only 19.4% of these patients.^9^^,^^10^ Although complete resolution of type B IMH with medical therapy has been reported in approximately 40% to 45% of cases,^14^^,^^15^ the presence of an ulcer-like projection or focal intimal disruption is associated with significantly higher rates of adverse aortic events and disease progression.^16^^,^^17^ Despite this high-risk feature, the dissection remained clinically uncomplicated, and medical management was continued given the absence of malperfusion, rupture, or hemodynamic instability on anti-impulse therapy.

Given the type I collagen deficiency in OI and its effects on vascular matrix integrity, progressive rather than resolving pathology might be expected after dissection.^5^^,^^18^ This favorable remodeling raises questions about phenotypic heterogeneity within OI. The cardiovascular phenotype in OI varies by subtype. Type I OI results from collagen type I alpha 1 haploinsufficiency, producing reduced quantities of structurally normal collagen, whereas types III and IV involve dominant-negative mutations that produce structurally abnormal collagen.^19^ Pediatric echocardiographic data have shown that aortic dilation is more pronounced in types III/IV, whereas type I patients demonstrate predominantly left ventricular dimensional changes without significant aortic dilation.^20^ The present patient had genetically confirmed type I OI. Whether this preserved collagen structure contributed to the favorable aortic remodeling observed in this case remains uncertain, though echocardiographic data showing less pronounced aortic dilation in type I compared with types III/IV suggest subtype-dependent differences in vascular involvement.^20^

## Conclusions

This case demonstrates complete resolution of acute uncomplicated TBAD with a thrombosed false lumen in the setting of a heritable collagen disorder, achieved with strict anti-impulse therapy and close surveillance. Future studies with molecular characterization, advanced imaging biomarkers, and long-term follow-up may better delineate which OI patients are at highest risk and which can be managed medically.

## Funding

This research received no specific grant from any funding agency in the public, commercial, or not-for-profit sectors.

## Declaration of generative AI and AI-assisted technologies in the writing process

During the preparation of this work, the authors used OpenEvidence to improve readability and scientific language of the case report. After using this tool, the authors reviewed and edited the content as needed and take full responsibility for the content of the publication.

## Disclosures

None of the authors have any conflicts of interest to disclose.
